# Is dying in hospital better than home in incurable cancer and what factors influence this? A population-based study

**DOI:** 10.1186/s12916-015-0466-5

**Published:** 2015-10-09

**Authors:** Barbara Gomes, Natalia Calanzani, Jonathan Koffman, Irene J. Higginson

**Affiliations:** King’s College London, Cicely Saunders Institute, Department of Palliative Care, Policy and Rehabilitation, Bessemer Road, SE5 9PJ London, UK; University of Edinburgh, Medical School, Centre for Population Health Sciences, Teviot Place EH8 9AG, Edinburgh, UK

**Keywords:** Bereavement, Health care surveys, Home care services, Neoplasms, Pain, Palliative care

## Abstract

**Background:**

Studies show that most patients with advanced cancer prefer to die at home. However, not all have equal chances and the evidence is unclear on whether dying at home is better. This study aims to determine the association between place of death, health services used, and pain, feeling at peace, and grief intensity.

**Methods:**

Mortality follow-back study of 352 cancer patients who died in hospital (n = 177) or at home (n = 175) in London, UK. Bereaved relatives identified from death registrations completed a questionnaire including validated measures of patient’s pain and peace in the last week of life and their own grief intensity. We determined factors influencing death at home, and associations between place of death and pain, peace, and grief.

**Results:**

Where people died was, for most (80 %), the place where they lived during their last week of life. Four factors explained >91 % of home deaths: patient’s preference, relative’s preference, home palliative care, or district/community nursing. The propensity of death at home also increased when the relative was aware of incurability and the patient discussed his/her preferences with family. Dying in hospital was associated with more hospital days, fewer general practitioner (GP) home visits, and fewer days taken off work by relatives. Adjusting for confounders, patients who died at home experienced similar pain levels but more peace in their last week of life (ordered log odds ratio 0.69, *P =* 0.007). Grief was less intense for their relatives than for those of patients who died in hospital (*β*, –0.15 around time of death and –0.14 at questionnaire completion, *P* = 0.02).

**Conclusion:**

The study suggests that dying at home is better than hospital for peace and grief, but requires a discussion of preferences, GP home visits, and relatives to be given time off work.

**Trial registration:**

National Institute of Health Research (NIHR) Clinical Research Network Portfolio. UKCRN7041.

**Electronic supplementary material:**

The online version of this article (doi:10.1186/s12916-015-0466-5) contains supplementary material, which is available to authorized users.

## Background

Studies of patients with advanced cancer and the general public show that most people would prefer to die at home [1, 2]. In the UK, the US, and Canada, more appear to be realising this wish [3–5]. In Japan, Germany, Greece, and Portugal a trend towards institutionalised dying persists [6–9]. Despite differing trends, the most frequent location of death for those dying from cancer is hospital, with marked variations in the odds of home death depending on illness-related, individual, and environmental factors [10–12]. Furthermore, the most important aim towards the end of life is to ensure the best possible palliative outcomes. Population ageing and larger numbers of people dying from cancer represent a demographic imperative for healthcare systems to ensure a dignified death for all [13, 14].

There is some evidence showing better results on psychological, social, and holistic measures of well-being in the last weeks or days of life for patients dying at home compared to hospital [15]. However, findings on symptoms and family outcomes are inconsistent, particularly for two widely researched outcomes: pain and grief [16–19]. Tang [20] found that pain was more likely for patients who died at home (compared to hospital), while Escobar Pinzon et al. [21] found the opposite (even when accounting for confounders) and Jordhoy et al. [22] found no difference. Addington-Hall and Karlsen, in the *Regional Study of Care for the Dying* (RSCD) in the UK [18], reported higher levels of grief among relatives of patients who died at home (compared to elsewhere), while Wright et al. [19], in the *Coping with Cancer* study in the US, found that relatives of patients who died in hospital were more likely to develop prolonged grief disorder. Ringdal et al. [23], however, in a randomised controlled trial of home palliative care, observed no difference by place of death. In an earlier study in London, Parkes [16] had reported worse symptom control for patients who died at home as opposed to hospital.

In order to meet patient preference at no expense of the best possible outcomes, it is crucial to find out whether death at home is better for patients and families. This study aimed to determine the association between place of death, health services used, and pain, feeling at peace, and grief intensity. We first identified factors influencing the propensity of dying at home rather than in hospital, and then compared the three outcomes (pain, peace, and grief).

## Methods

### Design, setting, and sample

QUALYCARE is a case-control study that used a mortality follow-back postal survey methodology of bereaved relatives (UKCRN7041; protocol in [24]). This is a well-established method internationally and recommended by the UK National End of Life Care Strategy in 2008 [25]. It was deemed appropriate for this study given the large sample size required and the need to examine common time periods before death for all in the sample.

The study took place in four health districts in London covering 1.3 million residents. An ecological analysis of cancer home death rates and socioeconomic deprivation levels informed the choice of districts to capture variation (Additional file 1).

Participants were identified from death registrations and approached by the Office for National Statistics (ONS). These were persons that registered the death of people aged ≥18 years who died from cancer (ICD-10 codes C00-D48) during a 1-year period (March 5, 2009, to March 4, 2010) having lived in the study districts. Patients were excluded if a coroner registered the death, since coroners would not be able to provide sufficient information about the patient’s care and outcomes.

The eligible sample included all who died at home and a random sample of 150 who died in a National Health System non-psychiatric hospital in each district (or all when the eligible number was <150, which happened in the two smaller districts). Cases comprised persons who died at home and controls were persons who died in hospital. A sample of 350 patients was chosen to detect a minimum standardised difference of 0.30 (power 80 %, significance 0.05) and allow regression analyses with a maximum of 35 variables entered and 19 retained, following Altman’s recommendations (n/10 variables and square root of sample size, respectively) [26].

Potential participants were contacted in writing by the ONS on behalf of the research team 4–10 months after registering the patient’s death, and asked to complete a structured postal questionnaire. Up to two reminders were sent to people who did not respond at 2 and 4 weeks after the initial posting; the second reminder included another copy of the questionnaire. In addition, pseudo-anonymised death registration data were provided by the ONS.

### Case ascertainment

Information on place of death is systematically recorded in death registration files, as reported by the informant (i.e. person who registers the death) within 5 days after the death occurred. We validated this information against questionnaire data. There were only two disagreements (Additional file 2). We also asked the respondents how long the patient had been in the place where they died.

### Measurement of factors and outcomes associated with home death

Our approach to measuring factors and outcomes associated with home death was guided by systematic reviews, a theoretical model of home death in cancer [11], and findings from the pilot preceding the study [27]. Additional files 3 and 4 include details of the factors and outcomes analysed; the information was collected in the same manner for cases and controls. We used validated specific health outcome measures: Palliative care Outcome Scale (POS) items to measure pain interference or affect on the patient (item ranging 0–4, higher scores meaning more pain interference) and the overall grade of peace during the last week of life (ranging 0–5, higher scores meaning more frequent feeling at peace) [28, 29]; and the Texas Revised Inventory of Grief (TRIG) to measure the respondent’s own grief intensity around time of death (TRIG I; reported retrospectively; scale ranging 8–40, higher scores meaning greater grief intensity) and at questionnaire completion (TRIG II; scale ranging from 13–65) [30]. Cronbach’s alpha estimates were 0.86 (TRIG I) and 0.93 (TRIG II) in the study. According to Bland and Altman [31], this demonstrates good internal consistency for comparing groups (α values of 0.70 to 0.80 are regarded as satisfactory for this purpose).

### Approvals and ethics

A National Health System research ethics committee (REC), the London – Dulwich REC, formerly King’s College London REC, approved the study (ref: 09/H0808/85). Together with the invitation letter and questionnaire, potential participants were mailed the study information sheet, a reply slip to decline participation, and a bereavement information leaflet. Return of a completed questionnaire was taken as informed consent. We followed piloted procedures for dealing with queries and distress [32] and checked returned questionnaires for participants requiring follow-up action (particularly answers to grief-related questions). If concerns arose and participants had agreed to be contacted, they were informed about local sources of support [24]. A data access agreement with the ONS governed the handling of death registration data.

### Analysis

#### Factors

We developed a multivariate model of factors associated with home death in two stages. Firstly, direct logistic regression tested a baseline model with nine variables covering all groups of factors proposed in the theoretical model: illness related, individual, and environmental (Additional file 5) [24]. Secondly, one-by-one, we added detail on cancer type and other variables associated with home death with *P* <0.250 in bivariate analysis, retaining those that stayed significant (*P* <0.050). Independent variables with near-zero cells and multicollinearity that cause quasi-separation of the dependent variable lead to mathematical problems in logistic regression rendering instability to models [33], and were therefore excluded. Variables with >10 % missing data were also excluded.

A sub-group analysis tested the final model only for patients who preferred to die at home. This aimed to help clinicians make decisions when caring for patients they know wish to die at home.

Finally, a sensitivity analysis examined the impact of non-response on the association of home death with five variables (available through death registrations for participants and non-participants): patient’s gender and age, country of birth, cancer type, and deprivation (measured using the Index of Multiple Deprivation 2010, in quintiles referring to the area of last residence of the deceased; more details in the footnote of Table 1 [34].

**Table 1:** Sample characteristics

	All	Home death	Hospital death
	(n = 352)	(n = 175)	(n = 177)
Patient’s gender			
Male	192 (54.5 %)	94 (53.7 %)	98 (55.4 %)
Female	160 (45.5 %)	81 (46.3 %)	79 (44.6 %)
Patient’s age			
Median in years (IQR)	76 (67–83)	76 (66–83)	76 (67–83.5)
Type of cancer (underlying cause of death)			
Digestive	105 (29.8 %)	59 (33.7 %)	46 (26.0 %)
Respiratory and intra-thoracic organs	80 (22.7 %)	40 (22.9 %)	40 (22.6 %)
Eye, brain, and other parts of the central nervous system	13 (3.7 %)	11 (6.3 %)	2 (1.1 %)
Breast	18 (5.1 %)	9 (5.1 %)	9 (5.1 %)
Lymphoid, haematopoietic, and related tissue	26 (7.4 %)	8 (4.6 %)	18 (10.2 %)
Genitourinary	67 (19.0 %)	25 (14.3 %)	42 (23.7 %)
Unspecified and other	43 (12.2 %)	23 (13.1 %)	20 (11.3 %)
Mobility at 3 months to death (EQ-5D)			
No problems	95 (28.4 %)	42 (25.6 %)	53 (31.0 %)
Some problems	211 (63.0 %)	106 (64.6 %)	105 (61.4 %)
Confined to bed	29 (8.7 %)	16 (9.8 %)	13 (7.6 %)
Self-care at 3 months to death (EQ-5D)			
No problems	148 (44.7 %)	68 (42.2 %)	80 (47.1 %)
Some problems	128 (38.7 %)	68 (42.2 %)	60 (35.3 %)
Unable to wash/dress her/himself	55 (16.6 %)	25 (15.5 %)	30 (17.6 %)
Usual activities at 3 months to death (EQ-5D)			
No problems	76 (22.8 %)	31 (18.8 %)	45 (26.6 %)
Some problems	152 (45.5 %)	82 (49.7 %)	70 (41.4 %)
Unable to perform usual activities	106 (31.7 %)	52 (31.5 %)	54 (32.0 %)
Patient’s country of birth			
UK/Ireland	288 (81.8 %)	142 (81.1 %)	146 (82.5 %)
Elsewhere	64 (18.2 %)	33 (18.9 %)	31 (17.5 %)
Patient’s ethnicity			
White British/Irish	291 (84.6 %)	145 (84.8 %)	146 (84.4 %)
White other/unspecified	21 (6.1 %)	13 (7.6 %)	8 (4.6 %)
Other	32 (9.3 %)	13 (7.6 %)	19 (11.0 %)
IMD 2010 (patient’s residence area) ^a^			
5^th^ quintile (least deprived)	89 (25.3 %)	46 (26.3 %)	43 (24.3 %)
4^th^ quintile	69 (19.6 %)	41 (23.4 %)	28 (15.8 %)
3^rd^ quintile	55 (15.6 %)	31 (17.7 %)	24 (13.6 %)
2^nd^ quintile	85 (24.1 %)	35 (20.0 %)	50 (28.2 %)
1^st^ quintile (most deprived)	54 (15.3 %)	22 (12.6 %)	32 (18.1 %)
Patient’s marital status			
Married/with partner	199 (58.0 %)	117 (68.8 %)	82 (47.4 %)
Widowed	86 (25.1 %)	34 (20.0 %)	52 (30.1 %)
Divorced/separated	25 (7.3 %)	9 (5.3 %)	16 (9.2 %)
Never married	33 (9.6 %)	10 (5.9 %)	23 (13.3 %)
Patient’s living with relatives			
Yes	249 (71.6 %)	145 (83.8 %)	104 (59.4 %)
No	99 (28.4 %)	28 (16.2 %)	71 (40.6 %)
Patient’s preference for PoD			
Home	262 (83.7 %)	168 (97.7 %)	94 (66.7 %)
Other or no preference	51 (16.3 %)	4 (2.3 %)	47 (33.3 %)
Relative’s gender			
Male	118 (33.8 %)	53 (30.6 %)	65 (36.9 %)
Female	231 (66.2 %)	120 (69.4 %)	111 (63.1 %)
Relative’s age			
Median in years (IQR)	59 (49–70)	60 (50–71)	57 (49–68)
Relative’s relationship to patient			
Spouse/partner	147 (41.9 %)	85 (48.6 %)	62 (35.2 %)
Son/daughter	142 (40.5 %)	75 (42.9 %)	67 (38.1 %)
Brother/sister	20 (5.7 %)	4 (2.3 %)	16 (9.1 %)
Other	42 (12.0 %)	11 (6.3 %)	31 (17.6 %)
Relative’s preference for PoD at 3 months to death			
Home	215 (61.8 %)	159 (91.9 %)	56 (32.0 %)
Other or no preference	133 (38.2 %)	14 (8.1 %)	119 (68.0 %)
Change in relative’s preference for PoD in 3 months prior death			
Yes	42 (12.7 %)	16 (9.5 %)	26 (16.0 %)
No	289 (87.3 %)	153 (90.5 %)	136 (84.0 %)

^a^ Index of Multiple Deprivation (IMD) 2010 national quintile of the area of last residence of the deceased. This information was provided by the Office for National Statistics based on death registration information of the patient’s lower layer super output area of residence (LSOA). The IMD 2010 score is a measure based on 38 indicators, chosen to cover a broad range of economic, social, and housing issues, into a single relative deprivation score for each LSOA in England [34]. The different domains are combined using appropriate weights to calculate the IMD 2010 score, an overall measure of multiple deprivation experienced by people living in the respective area. This represents unmet needs caused by lack of resources of all kinds (income, employment, health and disability, education skills and training, barriers to housing and other services, crime and living environment)

Percentages may not add to 100 % due to rounding

IQR, Interquartile range; PoD, Place of death

#### Outcomes

Multivariate ordinal regression (for pain and peace; non-normally distributed) and linear regression (for grief) determined associations with place of death. Potential confounders were entered and retained if significant (*P* <0.050), alongside factors associated with home death in our final model.

The models included the variable district to control for any design effect due to stratified sampling. When >10 % of individuals were excluded from the model due to missing data, χ^2^ tests identified any differential by place of death. Analyses were conducted in SPSS for Windows (version 19.0).

## Results

### Response rates and sample characteristics

We sampled 881 patients; 366 constituted cases and 515 were controls in the case–control study (flowchart in Additional file 6). Related to this sample, 352 bereaved relatives (40.0 %) completed the questionnaire. They were contacted a median of 232 days after the patient died, i.e. 7 months (interquartile range (IQR), 189–280 days), with no significant difference between cases and controls.

Patients died at a median age of 76 years (IQR, 67–83) and 55 % were men (Table 1). The two most common cancer groups were digestive (35 % of men, 23 % of women) and respiratory or intra-thoracic (21 % of men, 25 % of women). Most of the proxy respondents (66 %) were women and 90 % were first line relatives of the patient (i.e. spouses, partners, offspring, siblings, or parents). Their median age was 59 years (IQR, 49–70). Home death was preferred by >66 % of patients in both groups and 92 % of relatives of patients who died at home, but only by 32 % of relatives of patients who died in hospital, with <17 % of relatives in both groups changing their mind in the patient’s last 3 months of life.

**Fig. 1:**
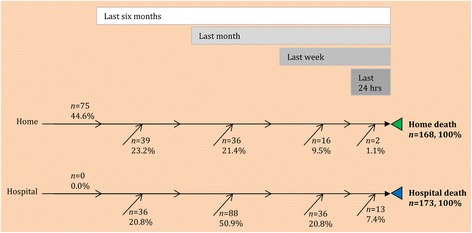
Permanence in place versus timing of last transition by place of death. The figure shows how long the patients were in the place where they died. Numbers and percentages by place of death are placed backwards from death according to the time period when the last transition happened. For example, 75 out of 168 people who died at home (45 %) were at home for 6 months or more (with no transition). Two (1 %) went home in their last 24 hours of life. Eleven patients had missing data: seven home deaths, four hospital deaths (including two people who gave inconsistent information).

For most decedents (80 %), where they died was also where they lived during their last week of life (Fig. 1). Of those who died in hospital, 28 % were admitted in the last week of life, 51 % in the last month but prior to the last week, and 21 % >1 month before they died. Of those who died at home, 45 % had not been admitted for the whole of their last 6 months of life and 11 % had gone home in their last week.

### Factors associated with dying at home rather than in hospital

**Fig. 2:**
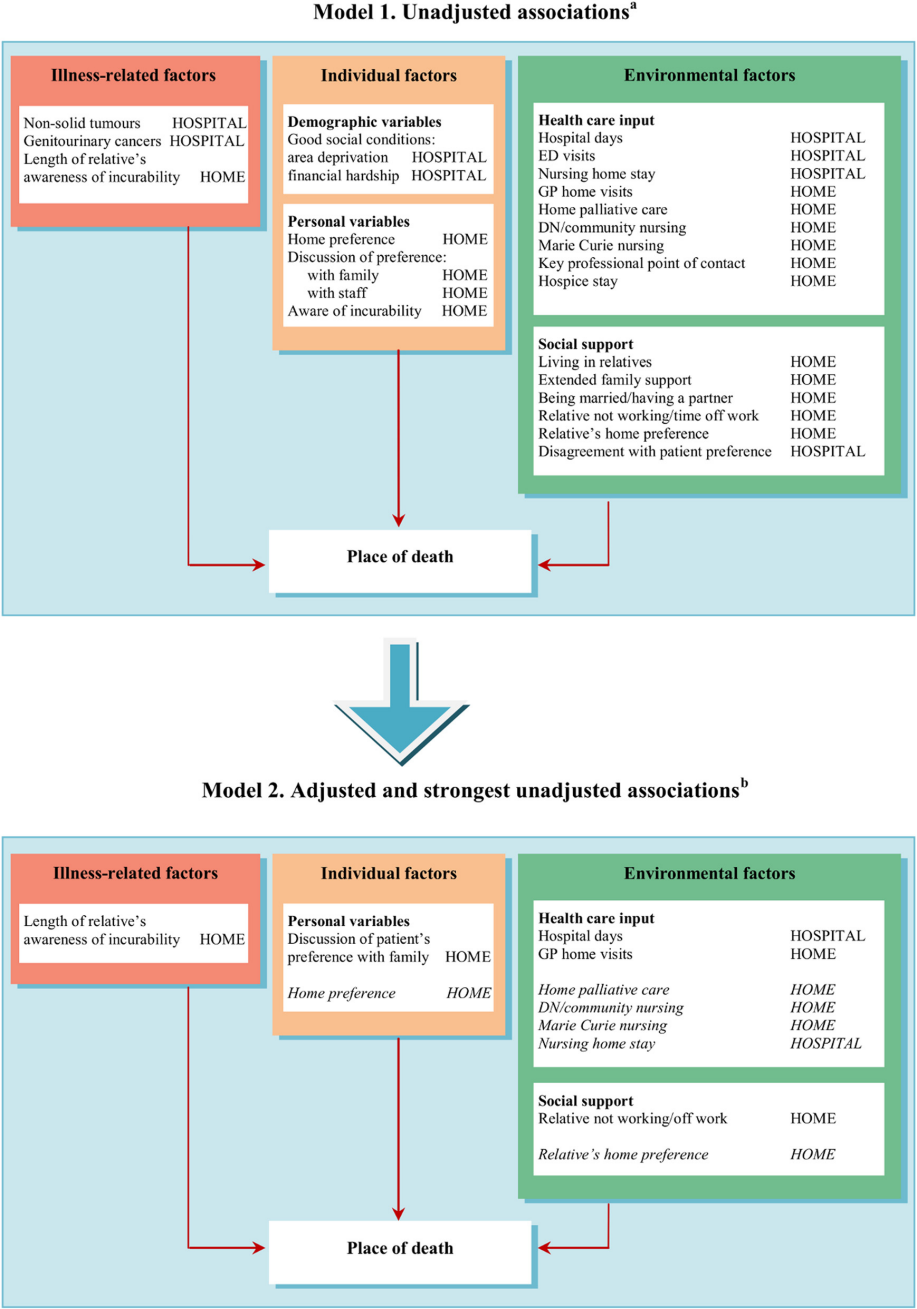
Unadjusted and adjusted associations with death at home. ^a^ Factors identified through bivariate analysis (*P* <0.05); ^b^ Factors retained in the final multivariate model (*P* <0.05) except for those in italic, which show the strongest unadjusted associations but due to near-zero cell frequencies and multicollinearity with place of death could not be included in the logistic regression model

Figure 2 summarises the associations contrasting bivariate and multivariate results. The first model includes unadjusted associations found through bivariate analysis (Additional file 7) and the second model includes adjusted but also the strongest unadjusted associations that due to near-zero cell frequencies and multicollinearity could not be included in multivariate logistic regression. Four inter-related factors explained >91 % of home deaths and >67 % of hospital deaths: preference for home death by the patient, preference for home death by the patient’s relative, receipt of home palliative care in the last 3 months of life, receipt of district/community nursing in the last 3 months of life. In addition, patients who died in hospital were less likely to have had Marie Curie nursing (these nurses care for people at home in the last few months or weeks of their lives, with the core service being one-to-one overnight nursing; 4 % in the hospital group compared to 41 % in the home group). Only seven patients who received care from Marie Curie nurses died in hospital. Three other variables had >10 % missing data and were therefore excluded from further analysis (whether the patient discussed their preference for place of death with professionals, whether the patient’s preference for place of death differed from the relative’s preference, and whether there had been a key professional point of contact in the 3 months before death).

**Fig. 3:**
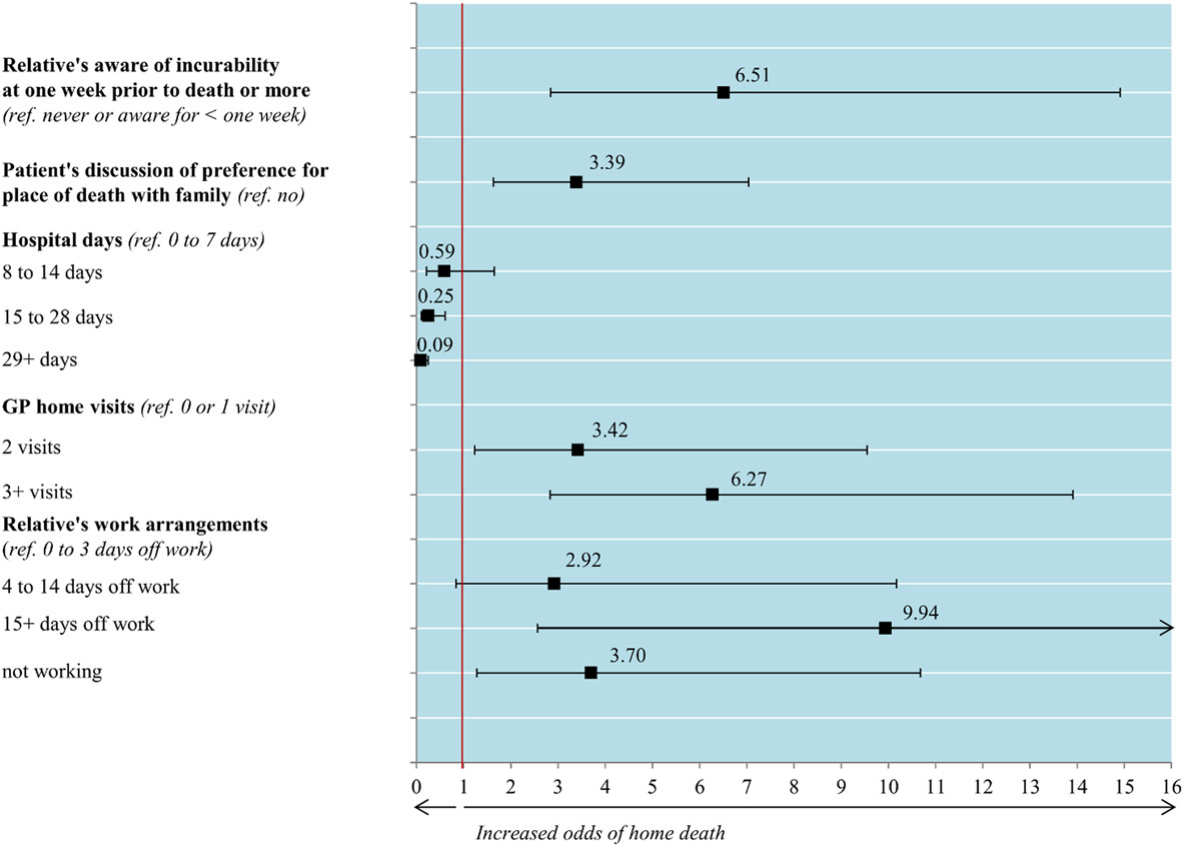
Five factors independently associated with death at home rather than in hospital. Factors retained in the final multivariate model (*P* <0.050). The dots present adjusted odds ratios and horizontal lines indicate 95 % confidence intervals. The model was statistically reliable [Hosmer and Lemeshow χ^2^(8,263) = 4.721, *P* = 0.787]; it correctly classified 82 % of the cases (persons who died at home) and 79 % of the controls (persons who died in hospital)

Figure 3 presents the relative influence of five factors in multivariate analysis. Two originated from the baseline model: hospital days and general practitioner (GP) home visits. Three represent new factors: length of relative’s awareness of incurability, discussion of patient’s preference for place of death with family, and relative’s work arrangements in the last 3 months before the patient died. All five associations were statistically significant (*P* ≤0.001), although there was a differential in missing data, with more information missing in the hospital group than in the home group (31 % and 20 %, *P* = 0.023).

A gradient was evident in the relationships of home death with hospital days and GP home visits (Fig. 3). When patients stayed 15–28 days in hospital during the last 3 months of life, the odds of dying at home were 75 % lower compared to patients with 0–7 days in hospital (adjusted odds ratio (AOR), 0.25; 0.10–0.61). Furthermore, the odds decreased by 91 % for patients who were in hospital for more than 28 days in the last 3 months of life (AOR, 0.09; 0.03–0.25). Patients who received three or more GP home visits during their last 3 months of life had six times greater odds of dying at home compared to patients with just one or no GP home visits (AOR, 6.27; 2.83–13.91). Those with two visits also had greater odds of dying at home (AOR, 3.42; 1.23–9.55). Home death odds were higher when relatives knew of incurability >1 week before death (AOR, 6.51; 2.84–14.91), when patients discussed their preferences with family (AOR, 3.39; 1.63–7.04), and if relatives took >14 days off work in the last 3 months before the patient died (AOR, 9.94; 2.56–38.65 versus <4 days off) or were not working (AOR, 3.70; 1.28–10.68).

**Table 2:** Multivariate ordinal regressions of factors associated with pain and peace

	Unadjusted				Adjusted		
	n	Median score (IQR)	Ordered log OR (95 % CI)	*P* value	n	Ordered log OR (95 % CI)	*P* value
**Pain in last week of life (POS item)**							
Place of death							
Hospital	167	2 (0–2)	*Ref.*		166	*Ref.*	
Home	168	1 (0–1)	−0.33 (–0.72 to 0.06)	0.094	168	−0.25 (–0.65 to 0.15)	0.228
Relative’s relationship to the patient							
Spouse/partner	139	1 (0–2)	*Ref.*		139	*Ref.*	
Son/daughter	135	1 (0–2)	0.39 (–0.04 to 0.82)	0.076	135	0.38 (–0.05 to 0.80)	0.087
Brother/sister	20	2 (1–2.5)	1.03 (0.19 to 1.88)	0.017	20	0.94 (0.08 to 1.80)	0.031
Other	40	1 (0–2)	0.47 (–0.17 to 1.10)	0.148	40	0.38 (–0.26 to 1.03)	0.245
**Peace in last week of life (POS item)**							
Place of death							
Hospital	161	3 (1.5–4)	*Ref.*		140	*Ref.*	
Home	169	4 (3–5)	0.93 (0.52 to 1.33)	<0.001	160	0.69 (0.19 to 1.19)	0.007
Relative’s relationship to the patient							
Spouse/partner	136	4 (3–4)	*Ref.*		124	*Ref.*	
Son/daughter	137	4 (3–4)	−0.14 (–0.57 to 0.29)	0.510	126	−0.16 (–0.62 to 0.30)	0.489
Brother/sister	15	3 (2–4)	−0.85 (–1.80 to 0.10)	0.080	12	−0.88 (–1.95 to 0.20)	0.112
Other	41	3 (1.5–4)	−0.75 (–1.38 to –0.12)	0.019	38	−0.51 (–1.17 to 0.16)	0.138
Length of relative’s awareness of incurability							
Never or aware for less than one week	76	3 (1–4)	*Ref.*		73	*Ref.*	
Aware for one week or more	237	4 (3–4)	0.65 (0.18 to 1.12)	0.006	227	0.69 (0.19 to 1.18)	0.389
Patient’s preference for place of death discussed with family							
No	122	4 (2–4)	*Ref.*		115	*Ref.*	
Yes	193	4 (3–4)	0.79 (0.38 to 1.21)	<0.001	185	0.64 (0.17 to 1.11)	0.008

POS items were used to measure pain interference or affect on the patient (0–4 scale, higher scores meaning more pain interference) and the overall grade of peace (0–5 scale, higher scores meaning more frequent feeling at peace) during the last week of life [28, 29]. The model on pain included 334 patients (94.9 %), with 167:1 patients per variable. Model statistics: Pearson χ^2^(24) = 19.281, *P* = 0.737; Nagelkerke R^2^ = 0.030. The model on peace included 300 patients (85.2 %), with 75:1 patients per variable. Model statistics: Pearson χ^2^(119) = 121.466, *P* = 0.420; Nagelkerke R^2^ = 0.121

CI, Confidence interval; OR, Odds ratio; POS, Palliative care Outcome Scale

**Table 3:** Multivariate linear regressions of factors associated with grief

	Unadjusted					Adjusted			
	n	M (SD)	*B* (95 % CI)	*β*	*P* value	*B* (95 % CI)	*β*	*P* value	s*r* ^2^
**Grief intensity around time of death (TRIG I)**									
Place of death									
Hospital	158	20.78 (8.25)	*Ref.*			*Ref.*			
Home	162	20.52 (7.91)	−0.25 (–2.03 to 1.52)	−0.02	0.779	−2.44 (–4.50 to –0.39)	−0.15	0.020	0.02
Relative’s relationship to the patient									
Spouse/partner	130	21.85 (8.16)	*Ref.*			*Ref.*			
Son/daughter	134	20.25 (7.58)	−1.61 (–3.55 to 0.33)	−0.10	0.104	−1.89 (–3.86 to 0.08)	−0.12	0.060	0.01
Brother/sister	15	17.87 (6.32)	−3.99 (–8.29 to 0.31)	−0.11	0.069	−4.83 (–9.09 to –0.56)	−0.13	0.027	0.02
Other	40	19.45 (9.40)	−2.40 (–5.25 to 0.45)	−0.10	0.098	−3.16 (–6.14 to –0.19)	−0.13	0.037	0.01
Relative’s presence at time of death									
No	94	19.04 (8.51)	*Ref.*			*Ref.*			
Yes	225	21.24 (7.72)	2.19 (0.27 to 4.12)	0.13	0.026	2.41 (0.28 to 4.54)	0.14	0.027	0.02
Patient’s preference for place of death discussed with family									
No	121	19.00 (8.01)	*Ref.*			*Ref.*			
Yes	188	21.57 (8.06)	2.57 (0.73 to 4.42)	0.16	0.006	3.01 (1.11 to 5.03)	0.19	0.002	0.03
**Grief intensity at time of questionnaire completion (TRIG II)**									
Place of death									
Hospital	158	43.11 (14.10)	*Ref.*			*Ref.*			
Home	158	45.02 (11.26)	1.91 (–0.92 to 4.73)	0.08	0.185	−3.63 (–6.76 to –0.50)	−0.14	0.023	0.01
Relative’s relationship to the patient									
Spouse/partner	126	49.21 (10.00)	*Ref.*			*Ref.*			
Son/daughter	133	41.92 (11.71)	−7.55 (–9.85 to –5.26)	−0.29	<0.001	−6.69 (–9.98 to –3.40)	−0.26	<0.001	0.04
Brother/sister	15	40.67 (16.17)	−10.93 (–15.75 to –6.21)	−0.19	<0.001	−6.78 (–13.45 to –0.11)	−0.11	0.046	0.02
Other	41	36.98 (16.18)	−11.92 (–15.22 to –8.63)	−0.31	<0.001	−12.54 (–17.09 to –7.99)	−0.32	<0.001	0.08
Relative’s presence at time of death									
No	90	38.77 (15.29)	*Ref.*			*Ref.*			
Yes	225	46.11 (10.94)	6.58 (4.36 to 8.81)	0.25	<0.001	4.77 (1.51 to 8.04)	0.17	0.004	0.01
Relative’s work arrangements in last three months of patient’s life									
0 to 3 days off work	58	37.50 (13.52)	*Ref.*			*Ref.*			
4 to 14 days off work	44	45.14 (11.92)	7.64 (2.88 to 12.40)	0.22	0.002	7.07 (2.41 to 11.73)	0.20	0.003	0.02
15+ days off work	47	50.32 (9.19)	12.82 (8.15 to 17.49)	0.37	<0.001	8.44 (3.64 to 13.23)	0.24	0.001	0.03
Not working	144	44.97 (12.37)	7.47 (3.77 to 11.18)	0.30	<0.001	3.87 (3.64 to 13.23)	0.15	0.048	0.01
Patient’s preference for place of death discussed with family									
No	123	41.83 (13.86)	*Ref.*			*Ref.*			
Yes	182	45.34 (11.96)	2.80 (0.52 to 5.09)	0.11	0.016	2.88 (0.07 to 5.82)	0.11	0.056	0.01

The TRIG was used to measure grief intensity for the respondent; the first scale asks respondents about their experience at the time of death (TRIG I 8–40 scale, 8 items, measured retrospectively) and the second scale asks about their experience at the time of questionnaire completion (TRIG II 13–65 scale, 13 items; higher scores meaning greater grief intensity; [30]). TRIG I model included 307 bereaved relatives (87.2 %), with more missing amongst hospital deaths than amongst home deaths (16.4 % vs. 9.1 %, *P* = 0.042). Model statistics: R for regression significantly different than zero, F(9,297) = 4.492, *P* <0.001; R^2^ = 0.120, adjusted R^2^ = 0.093. TRIG II model included 281 bereaved relatives (79.8 %), with no significant differences in the proportion missing amongst hospital deaths and home deaths (22.6 % vs. 17.7 %, *P* = 0.254). Model statistics: R for regression significantly different than zero, F(12,268) = 8.181, *P* <0.001; R^2^ = 0.268, adjusted R^2^ = 0.235

CI, Confidence interval; B, Unstandardised regression coefficient; β, Standardised regression coefficient; M, mean; s*r* ^2^, Semipartial correlation; SD, Standard deviation; TRIG, Texas Revised Inventory of Grief

### Sub-group and sensitivity analyses

Testing the five-factor model on patients who preferred to die at home (i.e. excluding 38 patients who preferred to die elsewhere and 13 who had no preference), all factors remained significant except for whether the patient discussed their preference for place of death with family. Sensitivity analysis examining the impact of non-response on the results showed no differences on the influence that the five factors available for this analysis had on home death (patient’s gender, age, cancer type, country of birth, and deprivation; analyses in Additional files 8 and 9).

### Differences in pain, peace, and grief by place of death

The results for pain and peace were maintained in adjusted analyses (Table 2). Based on respondents’ accounts, we found that the level of pain experienced by patients in their last week of life was no different for those who died at home than for those who died in hospital (median 1 (slightly, IQR, 0–2) compared to 2 (moderately, 0–2) in POS item ranging 0–4; M-W (335) = 15.456, *z* = 1.671, *P* = 0.095). Regardless of severity level, the prevalence of pain was 61.9 % among patients who died at home and 70.1 % among patients who died in hospital. Moderate to overwhelming pain was experienced by 53.3 % of patients in the hospital group as opposed to 44.0 % of those in the home group.

Patients who died at home were reported to feel more frequently at peace during their last week of life than patients who died in hospital (median 4 (most of the time, IQR, 3–5) compared to 3 (some of the time, 1.5–4) in POS item ranging 0–5, M-W(330) = 9.809, *z* = –4.546, *P* <0.001). A minority (11.8 % of patients who died at home and 24.8 % of those who died in hospital) were not very often or not at all at peace in their last week of life.

The grief intensity experienced by the respondents around time of death and at questionnaire completion (median of 7 months after the patient died) was similar in the home and hospital groups (mean, 20.52 (SD, 7.91) and 20.78 (8.25), respectively, in TRIG I scale ranging 8–40, *t*-test(318) = –0.281, *P* = 0.779; mean, 45.02 (11.26) and 43.11 (14.10), respectively, in TRIG II scale ranging 13–65, *t*-test(299) = 1.327, *P* = 0.185). However, once confounders were taken into account (stronger grief reactions were associated with spouses/partners, presence at time of death, family discussion of preferences for place of death and relative’s time off work in the last 3 months before the patient died), the levels of grief were less intense when the patient died at home compared to hospital, both at the time of death (*β* –0.15, *P* = 0.020) and questionnaire completion (*β* –0.14, *P* = 0.023; Table 3).

## Discussion

To date, QUALYCARE is the most comprehensive population-based study of factors and outcomes associated with dying at home compared to hospital; it includes over 350 people who died from cancer and their relatives in the largest metropolitan area in the UK. The study fills in a critical gap in providing new evidence suggesting that dying at home is better than hospital for peace and grief, with no difference in the pain level experienced in the last week of life.

### Limitations

There are limitations related to the study design, measurement, and analysis. The study is retrospective, and hence we show associations which do not necessarily indicate causality. It was conducted in London, therefore the transferability of findings to other regions where home care services (including palliative care) are less available, for example, is uncertain. The response rate was low (40 %), although it falls within the range of similar population-based postal surveys with bereaved relatives in the UK [35–37] and also compares to service-based surveys in the UK and Canada [38, 39]. Through sensitivity analysis, we identified no impact of non-response on the association of five variables with home death, although we were only able to examine a limited number of variables (those available for participants and non-participants).

The measurement of factors and outcomes was systematic and standardised but some variables (subjective factors, pain, peace) are vulnerable to recall and observer bias from respondents. Some studies suggest that relatives either overestimate or polarize their ratings of patient’s pain 4–7 months after death compared to prospective reports [40, 41]. Our data do not show a polarization, but we cannot confirm whether the levels of pain, for example, were overestimated (this would require prospective data on the same patients). Even so, the pain prevalence found (61.9 % among patients who died at home, 70.1 % among patients who died in hospital) was comparable to the pooled estimate of 64 % (95 % CI, 58–69 %) reported in a meta-analysis of advanced cancer patients, which excluded proxy data [42]. In contrast, it was lower than the weighted mean pain prevalence recently reported by Higginson et al. (76.1 % and 73.9 % including and excluding proxy data, respectively) [43]. These comparisons suggest that if there was overestimation, the magnitude of an effect on findings is likely to be small. The use of a postal method (which offers protection against social desirability) and the high salience of the events in question (circumstances surrounding the patient’s death) might have increased recall accuracy in both cases and controls.

### Factors associated with dying at home

We found several modifiable factors associated with place of death that are amenable to intervention and are rarely measured. Namely, we identified four conditions that are almost essential for patients to die at home rather than in hospital: patient’s preference, relative’s preference, receipt of home palliative care, and of district nursing/community nursing. The study also shows that, if patients get intensive nursing care specific to the end of life (provided by Marie Curie nurses), they very rarely die in hospital. However, 96 % of those who died in hospital did not get such help. The study observes dose-response relationships for two factors from a previous model [11]: hospital days and GP home visits. It challenges current thinking about the influence of patient’s functional status, social conditions, and living arrangements, showing no association once other factors are considered. Importantly, we identified three factors previously overlooked – length of relative’s awareness of incurability, discussion of patient’s preference with family, and relative’s work arrangements in the 3 months before death. Our final model explained well why some patients died at home whilst others died in hospital. Subject to testing, this may be effective for clinical decision-making.

### Preferences for place of death

Given the importance of meeting people’s preferences for place of death and preferences being one of the most influential factors on actual place of death, it is meaningful that home was reported as the preferred location of death for more than two thirds of patients in both groups. However, it should be noted that not all wish to die at home and that there are situations in which dying at home may not be feasible. Moreover, a home death was preferred by 92 % of relatives of patients who died at home, but only by 32 % of relatives of patients who died in hospital. Adding to findings from a systematic review of preferences for dying at home [1], we found a lower home preference from relatives compared to patients; this stresses the crucial role of families in caring for patients at home and in decision-making processes [1, 44]. Most relatives said that their own preferences were stable in the last 3 months before death, with less than a fifth in both groups (cases and controls) changing their mind during this period. Early conversations about preferences involving patients and families seem, therefore, appropriate, provided they are well-conducted and preferences are monitored over time.

### Differences in outcomes between dying at home and in hospital

Our findings suggest that, in addition to increasing choice, a home death is associated with similar experiences (in terms of pain) and sometimes better experiences and outcomes (in terms of peace and grief) for people dying of cancer and for their relatives. The study helps elucidate previous contradictory evidence on pain and grief, and provides some of the first evidence on sense of peace in relation to place of death.

#### Pain

The absence of a statistically significant difference in the levels of pain experienced by patients who died at home and by those who died in hospital concurs with some studies [22, 45] and disagree with others [16, 21]. Our findings differ from a seminal study conducted by Parkes in London (1967–1971) [16], where among a sample of 165 patients, he found significantly greater pain at home than in hospital. However, Parkes included in the home group people who were at home for most of the time but were admitted to hospital in their last week of life. As it is possible that admission could be due to problems controlling pain, the inclusion of this sub-group in home deaths (18/65 of the home group) may have shaped the results.

It is indeed plausible that some patients who died in hospital were admitted because of more severe problems controlling pain at home, while those who stayed at home might have experienced fewer complications in the last week of life. It is also plausible that relatives were more aware of pain when people were at home, hence our findings on pain must be regarded with care. However, it is of relevance that, in QUALYCARE, the median level of pain was lower in the home group than in the hospital group, though the difference did not reach statistical significance. Interestingly, one recent study that shares the design, measurement, and timeframe with QUALYCARE (the EPACS study in Germany) found that patients who died at home were less likely to have experienced pain (57.8 % vs. 67.9 %) than patients who died in hospital [21].

#### Peace

Few studies have examined whether patients’ sense of peace differs by place of death. Our findings support those from a small observational study in Philadelphia [46], where more bereaved relatives of patients who died at home thought these had been at peace than relatives of patients who died elsewhere (16/18 and 5/10, respectively, *P* = 0.003). In contrast, in the South West of the Netherlands, van der Heide et al. [45] found no differences in physicians’ perspectives on whether the patient died peacefully between those who died at home and elsewhere (29/42 and 28/41, respectively, *P* = 0.94). However, this finding is limited to the degree the physicians were involved and present in the last days of life (which was unknown).

The one point difference in median peace scores between the home and hospital groups (4, i.e. most of the time, compared to 3, i.e. some of the time, measured through the POS peace item with a 0–5 range) is of clinical significance. However, both medians were higher (i.e. better) than the threshold recommended by Selman et al. [47] as indicative of distress in the POS peace item, i.e. scores of 0–1. Applying this threshold, the differences are still evident: 11.8 % of patients who died at home and 24.8 % of those who died in hospital were on or below the recommended threshold, indicating distress.

It is possible that this difference reflects the importance of some of the dimensions that Selman et al. [47] identified in the concept of peace with patients. These include being aware and accepting death, but also relational aspects such as the ability to be at home with family. However, the association of home death with increased peace persisted when adjusted for relative’s awareness of incurability, family discussion of preferences for place of death, and respondent’s relationship to the patient. The finding of differences in peace but not in pain suggests the former may encompass but transcends the latter. Other aspects may play a role, for example, those related to the environment in hospitals (noisy and busy settings) and the patient’s consciousness level (e.g. being asleep may be regarded by the relatives as being peaceful).

#### Grief

Our findings showing less intense grief for relatives in the home group refute previous UK population-based data from the 1990’s [18]. RSCD investigators found that bereaved relatives of patients who died at home had higher levels of psychological distress around 10 months after the patient died as measured by the GHQ-28 (mean score 24.7 compared to 22.2, *P* <0.01) and were more likely to say they missed the deceased a great deal, and less likely to say that they had come to terms with the death, that they looked forward to things as usual, or that things were going reasonably well for them by then. The results on the two latter aspects were similar in the sub-group of spouses, whilst differences in the former two lost significance in this sub-group. No adjustments were made to the results, though, and this is important because relatives of patients who die at home are more likely to be spouses, but also more likely be present at the time of death, factors we found were associated with greater grief intensity. Adjusting for these confounders is, therefore, important.

In addition, the RSCD compared psychological distress and three items measuring cognitive and emotional aspects of grief. However, grief encompasses other important facets (e.g. behavioural, relational) [48]. In QUALYCARE, we measured the whole construct of grief, using a validated measure with high Cronbach’s alphas, demonstrating good internal consistency for the purpose of comparing groups [32]. However, it is more plausible that adjustments rather than measurement issues explain the differences between our results and the RSCD. It is also likely that home support increased over time (from 1990 to 2010), which may impact on grief adjustment.

Our findings concur with the *Coping with Cancer* study conducted more recently in the US [19], which showed that relatives of patients who died in hospital were at heightened risk of prolonged grief disorder (assessed using a validated scale) compared to relatives of patients who died at home with hospice care (AOR, 8.83; 95 % CI, 1.51–51.77). An important finding from the *Coping with Cancer* study was that none of the psychological variables measured at baseline (median 4.5 months before the patient died) were associated with place of death, which suggests pre-bereavement psychological morbidity may not confound the effect of place of death on post-bereavement complications. QUALYCARE results on grief also align with the *Cambridge Hospital at Home* randomised controlled trial in the UK [49].

### Implications for clinical practice

It is clear from the findings that most people dying of cancer prefer to die at home and that this wish is often supported by family, yet seldom met. Those that do die at home (in regions of London) appear to spend their last days in greater peace, nearly always with family around them, and with pain controlled to a similar level to those who die in hospital. Based on the data, nearly two fifths of the people who die of cancer at home in the regions in the study can expect a pain-free death. Around another fifth are affected only slightly – this makes over half of all home deaths in cancer. Such encouraging reality reflects multiple factors, which include the work of clinicians on the ground, particularly of those working in the community, to ensure that patients and families are adequately supported at home. Yet, the findings highlight the need to do more. There are some people for whom death at home is not peaceful and pain-free, and there are relatives that are left in intense grieving. Input from home palliative care services, district/community nurses, and GPs is essential, but not enough to guarantee to a patient with advanced cancer who wants to die at home that they will achieve their wish. Other factors in our model (Figs. 2 and 3) require attention from all clinicians involved in cancer care in terms of risk assessment and care management.

## Conclusions

QUALYCARE presents novel knowledge suggesting that dying at home is better than hospital for peace and grief, and it also shows what needs to be in place for advanced cancer patients to die at home if they wish. Examining the variations associated with home death in cancer in a comprehensive way revealed that dying at home is a complex yet tangible goal, one that can be achieved in comfortable conditions. The findings indicate that making this a reality for more patients with incurable cancer requires a wide response: from oncologists to check the identified risk factors and discuss preferences with patients and families, from GPs to be proactive and make home visits, and from policymakers to implement comprehensive home care packages and develop measures to sustain family involvement in care. Failure to do this may contribute to crises and subsequent admissions, leading to an undesired death in hospital, less peaceful than can be achieved at home and more difficult for relatives to live with.

## Additional files

Additional file 1:
**Ecological analysis of cancer home death rates and deprivation levels in London.** (DOCX 97 kb) Additional file 2:
**Discrepancies in place of death information: death registration versus questionnaire data.** (DOCX 11 kb) Additional file 3:
**Factors potentially associated with home death: source and coding.** (DOCX 30 kb) Additional file 4:
**Outcome measurement: pain, peace, and grief.** (DOCX 15 kb) Additional file 5:
**Model of death at home in cancer tested in the QUALYCARE study.** (DOCX 14 kb) Additional file 6:
**Recruitment flow diagram.** (DOCX 51 kb) Additional file 7:
**Bivariate analysis of factors associated with home death.** (DOCX 24 kb) Additional file 8:
**Sub-analysis of five-factor model with patients who preferred to die at home.** (DOCX 16 kb) Additional file 9:
**Sensitivity analysis of non-participation on factors associated with home death.** (DOCX 21 kb)

## Abbreviations

AOR: Adjusted odds ratio
GP: General practitioner
IQR: Interquartile range
NIHR: National Institute of Health Research
ONS: Office for National Statistics
POS: Palliative care Outcome Scale
RSCD: Regional Study of Care for the Dying
TRIG: Texas Revised Inventory of Grief
