# Supplementary material for: Is dying in hospital better than home in incurable cancer and what factors influence this? A population-based study
Source: BMC Med. 2015 Oct 9;13:235. doi: 10.1186/s12916-015-0466-5 (PMC4599664; doi:10.1186/s12916-015-0466-5)
Supplement: Additional file 1: — Ecological analysis of cancer home death rates and deprivation levels in London. (DOCX 97 kb) [file 12916_2015_466_MOESM1_ESM.docx]

**Additional File 1**

**Ecological analysis of cancer home death rates and deprivation levels in London**

By the time this analysis was undertaken, health districts were organised in Primary Care Trusts (PCTs). Home death rates for all registered cancer patients by PCTs were provided to us by the Thames Cancer Registry. By then, the latest data reported to 2005 [1]. Indices of Multiple Deprivation 2004 (IMD 2004) for the respective local authorities were obtained from the Neighbourhood Statistics website [2]. Home death rates were plotted against IMD 2004 average scores (Figure 1).

**Figure 1. Cancer home death percentages in 2005 by IMD 2004 average score, London**

district 3

district 1

district 2

district 4

Cancer home death rates for registered cancer patients in 2005 were provided by the Thames Cancer Registry. IMD 2004 scores for the respective local authorities were obtained from the Neighbourhood Statistics website [2]. IMD scores for district 3 and for one other district were averages of the scores of the local authorities covered by the districts.

Based on the scatter plot, we selected districts in the upper and lower terciles of cancer home death rates and deprivation scores, so that the study would cover the four extremes of the plot. Districts of large size (district 1 and 3) and geographically close (district 2 and 4) were preferred [3].

Cancer home death rates in 2005 were 25.8% in HR 1 and 14.9% in HR 3 (both relatively affluent areas), 21.7% in HR 2 and 13.9% in HR 4 (relatively deprived areas). Figure 1 shows the Pearson’s correlation coefficient and R^2^. Although there appeared to be a negative correlation between HRs’ cancer home death rates and deprivation scores, this was not strong or significant.

All the four districts chosen had key London features, such as young populations and high proportions of ethnic minorities compared with the rest of the country; but with a substantial degree of variation (Table 1). The percentage of people aged 65 years or over varied from 10.2% in district 2 to 16.9% in district 1. The percentage of non-white British/Irish was lowest in district 1(11.9%) and highest in district 4 (47.9%).

There were other distinguished characteristics, namely in the provision of specialist palliative care services in 2009-10 [4]. For example, a large hospital serves district 3 (a large district with a relatively low cancer home death rate), where one hospice was the sole provider of home palliative care, supported by a hospital to home service at the hospital since 2007. District 1 (a large district with a relatively high cancer home death rate) is in the catchment area of two recently merged hospice services, both of which are high capacity services providing 24/7 palliative care at home. District 2 is the smallest of the four districts (with a relatively high cancer home death rate), which in 2009 was served by one specialist palliative care team offering home care (based at a local hospital) and two hospices providing home care. District 4 (also a small district but with a low cancer home death rate) was in the catchment area of a Hospice at Home service and community Macmillan nursing teams.

**Table 1. Four health districts in QUALYCARE**

|  | **District 1** | **District 2** | **District 3** | **District 4** |
| --- | --- | --- | --- | --- |
| **CENSUS 2001 information**^a^ |  |  |  |  |
| Residents | 295,532 | 175,797 | 367,676 | 181,286 |
| % aged 65+ | 16.9% | 10.2% | 13.7% | 12.4% |
| % white British/Irish | 88.1% | 62.5% | 76.2% | 52.1% |
| **Information at sampling stage** |  |  |  |  |
| IMD 2004 average score^b^ | 13.17 | 42.65 | 13.89 | 31.68 |
| % cancer home deaths 2005^c^ | 25.8% | 21.7% | 14.9% | 13.9% |
| **Information at study stage** |  |  |  |  |
| IMD 2010 average score^b^ | 14.95 | 35.87 | 15.00 | 24.59 |
| % cancer home deaths 2009-10^d^ | 27.5% | 17.8% | 19.5% | 25.4% |

^a^ Obtained from the Neighbourhood Statistics website [2].

^b^ IMD scores for district 3 are the average of the two local authorities.

^c^ Refers to the year of 2005, data provided by the Thames Cancer Registry on all registered patients.

^d^ Refers to the exact time period for sampling (March 2009 to March 2010), data provided by the Guidecare Project on all cancer registered deaths (2012).

**References**

1. Madden P, Coupland V, Moller H, et al. Exploring methods to identify London primary care trusts with low proportions of deaths from cancer at home or in hospice in the period 2002-2005 [poster],  Cancer in South East England 2006: cancer incidence, prevalence, survival and treatment for residents of South East England, London: King's College London, Thames Cancer Registry, 2008.

2. ONS: Neighbourhood Statistics. http://www.neighbourhood.statistics.gov.uk (2004).

3. Madden P, Coupland V, Moller H , Davies E. Using maps and funnel plots to explore variation in place of death from cancer within London, 2002-2007. Palliat Med 2011; 25(4) 323-32.

4. Help the Hospices. Hospice and palliative care directory: United Kingdom and Ireland 2009-10, London: Help the Hospices; 2009.
