# Supplementary material for: Is dying in hospital better than home in incurable cancer and what factors influence this? A population-based study
Source: BMC Med. 2015 Oct 9;13:235. doi: 10.1186/s12916-015-0466-5 (PMC4599664; doi:10.1186/s12916-015-0466-5)
Supplement: Additional file 2: — Discrepancies in place of death information: death registration versus questionnaire data. (DOCX 11 kb) [file 12916_2015_466_MOESM2_ESM.docx]

**Additional File 2**

**Discrepancies in place of death information: death registration versus questionnaire data**

| **Case number** | **Place of death** | | **Details** | **Final coding** |
| --- | --- | --- | --- | --- |
|  | **Death registration** | **Survey questionnaire** |  |  |
| 1 | hospice | own home | Analysis of the full questionnaire indicated that the patient died at home; the respondent (husband) stated the patient died at home and did not stay in hospice. | home |
| 2 | hospital | own home | The respondent (brother-in-law) stated the patient died at home but said the last hospital admission was to the same hospital as in death registration. | home |
