# Supplementary material for: Is dying in hospital better than home in incurable cancer and what factors influence this? A population-based study
Source: BMC Med. 2015 Oct 9;13:235. doi: 10.1186/s12916-015-0466-5 (PMC4599664; doi:10.1186/s12916-015-0466-5)
Supplement: Additional file 3: — Factors potentially associated with home death: source and coding. (DOCX 30 kb) [file 12916_2015_466_MOESM3_ESM.docx]

**Additional File 3**

**Factors potentially associated with home death: source and coding**

**Table 1. Illness-related and individual factors**

|  | **Details** | |  | **Bivariate analysis** | |
| --- | --- | --- | --- | --- | --- |
| **Variables** | **Source** | **Original coding** |  | **Coding/recoding** | **Test used** |
| type of cancer (underlying cause of death) | death registration | ICD-10 codes (free-text) |  | digestive  respiratory and intra-thoracic organs  eye, brain and other parts of the CNS  breast  lymphoid, haematopoietic and related tissue  genitourinary  unspecified and other | Fisher’s exact test and post-hoc *X*^2^ tests for each of the seven main types vs. others |
| mobility at 3 months to death (EQ-5D) | survey | no problems (1)  some problems (2)  confined to bed (3) |  | no problems (1)  some problems (2)  confined to bed (3) | MWU |
| self-care at 3 months to death (EQ-5D) | survey | no problems (1)  some problems (2)  unable to wash or dress her/himself (3) |  | no problems (1)  some problems (2)  unable to wash or dress her/himself (3) | MWU |
| usual activities at 3 months to death (EQ-5D) | survey | no problems (1)  some problems (2)  unable to perform usual activities (3) |  | no problems (1)  some problems (2)  unable to perform usual activities (3) | MWU |
| length of illness | survey | a day or more, but less than a week  one week or more, but less than one month  one month or more, but less than six months  six months or more, but less than one year  one year or more, but less than three years  three years or more |  | less than six months  six months or more, but less than one year  one year or more, but less than three years  three years or more | MWU |
| length of relative’s awareness of incurability | survey | never aware  aware for a day or more, but less than a week  one week or more, but less than one month  one month or more, but less than six months  six months or more, but less than one year  one year or more, but less than three years  three years or more |  | never aware or aware for less than one week  aware for one week or more | *X* ^2^ |
| patient’s gender | death registration | man/woman |  | man/woman | *X* ^2^ |
| patient’s age | death registration | years |  | 20-49  50-59  60-69  70-79  80-89  90+ | MWU |
| patient’s country of birth | death registration | 81 countries (free-text) |  | UK/Ireland  others | *X* ^2^ |
| patient’s ethnicity | survey | white British  white other (specify)  black Caribbean  black African  black other (specify)  Indian  Pakistani  Bangladeshi  Chinese  other ethnic group (specify) |  | white British/Irish  (including when British or Irish were mentioned in ‘white other ‘category)  white other/unspecified  (including when white was mentioned in ‘other ethnic group‘ category)  other | *X* ^2^ |
| patient’s financial hardship | survey | living comfortably (1)  doing alright (2)  just about getting by (3)  finding it quite difficult (4)  finding it very difficult (5) |  | living comfortably (1)  doing alright (2)  just about getting by (3)  finding it difficult (4) | MWU |
| Index of Multiple Deprivation (IMD) 2010 | death registration^a^ | score and quintiles:  5^th^ quintile (least deprived)  4^th^ quintile  3^rd^ quintile  2^nd^ quintile  1^st^ quintile (most deprived) |  | quintiles:  5^th^ quintile (least deprived)  4^th^ quintile  3^rd^ quintile  2^nd^ quintile  1^st^ quintile (most deprived) | MWU |
| patient’s preference for place of death | survey | own home  home of relative or friend  hospice  hospital  nursing home  residential home  elsewhere (*specify*)  did not have a preference |  | home preference  *(including own home, home of relative or friend, and when home was mentioned in ‘elsewhere’ category alongside other places)*  other or no preference | *X*^2^ |
| discussion of preference with family | survey | yes/ no |  | yes/ no | *X*^2^ |
| discussion of preference with professional | survey | yes/no |  | yes/ no | *X*^2^ |
| patient’s awareness of incurability | survey | certainly knew  probably knew  probably did not know  definitely did not know |  | certainly knew  probably knew  probably did not know  definitely did not know | MWU |
| patient’s religion | survey | no religion  Christian  Buddhist  Hindu  Jewish  Muslim  Sikh  other (*specify*) |  | no religion  Christian *(including when mentioned in ‘other’)*  other religion | *X*^2^ |

^a^ Scores and classification in national quintiles provided by the Office for National Statistics, based on death registration information of the patient’s lower layer super output area of residence.

MWU – Mann-Whitney U-test; *X*^2^ – Chi-squared test

**Table 2. Environmental factors**

|  | **Details** | |  | **Bivariate analysis** | |
| --- | --- | --- | --- | --- | --- |
| **Variables** | **Source** | **Original coding** |  | **Coding/recoding** | **Test used** |
| Emergency Department visits in last 3 months of life | survey | yes/no and number of visits |  | 0 visits  1 visit  2 visits  3+ visits | MWU |
| hospital days in last 3 months of life | survey | yes/no and number of days |  | 0 to 7 days  8 to 14 days  15 to 28 days  29+ days | MWU |
| nursing home stay in last 3 months of life | survey | yes/no and number of days |  | yes/no | *X*^2^ |
| hospice stay in last 3 months of life | survey | yes/no and number of days |  | yes/no | *X*^2^ |
| General practitioner (GP) home visits in last 3 months of life | survey | yes/no and number of visits |  | 0 or 1 visit  2 visits  3+ visits | MWU |
| home palliative care in last 3 months of life^a^ | survey | yes/no and number of contacts |  | yes/no | *X*^2^ |
| Marie Curie nursing in last 3 months of life^b^ | survey | yes/no and number of contacts |  | yes/no | *X*^2^ |
| DN/community nursing in last 3 months of life^b^ | survey | yes/no and number of contacts |  | yes/no | *X*^2^ |
| help from home care workers^b^ | survey | yes/no and number of hours/week |  | yes/no | *X*^2^ |
| key professional point of contact | survey | yes/no |  | yes/no | *X*^2^ |
| living in relatives | survey |  |  | yes/no | *X*^2^ |
| family caregivers | survey | item on whether the respondent was involved in care (yes/no) and item on whether other family or friends were involved (yes/no and if yes how many excluding respondent – one person only, two persons, three persons, four persons, five or more persons) |  | 0 or caregiver  2 or 3 caregivers  4+ caregivers | MWU |
| patient’s marital status | survey | married/with partner  widowed  divorced  separated  never married |  | married/with partner  widowed  divorced/separated  never married | *X*^2^ |
| relative’s work arrangements in last 3 months of life | survey | whether the respondent stop working or reduced work (yes, no was retired, no was unemployed, no was studying, no carried on working equal hours), and if yes, number of days off work |  | 0 to 3 days off work  4 to 14 days off work  15+ days off work  not working | MWU |
| relative’s preference for place of death at 3 months before death | survey | own home  home of relative or friend  hospice  hospital  nursing home  residential home  elsewhere (*specify*)  did not have a preference |  | home preference  *(including own home, home of relative or friend, and when home was mentioned in ‘elsewhere’ category alongside other places)*  other or no preference | *X*^2^ |
| change in relative’s preference | survey | yes/no |  | yes/no | *X*^2^ |
| relative’s disagreement with patient preference | survey | computed from items on patient and relative’s preferences for place of death |  | yes/no | *X*^2^ |

^a^ This included palliative care teams, Macmillan nurses, staff described as hospice or palliative care by the respondents in items to report other nurses and other professionals. Numbers of contacts were summed.

^b^ This included references by respondents in items to report other nurses and professionals.

MWU – Mann-Whitney U-test; *X*^2^ – Chi-squared test
