# Supplementary material for: Is dying in hospital better than home in incurable cancer and what factors influence this? A population-based study
Source: BMC Med. 2015 Oct 9;13:235. doi: 10.1186/s12916-015-0466-5 (PMC4599664; doi:10.1186/s12916-015-0466-5)
Supplement: Additional file 4: — Outcome measurement: pain, peace, and grief. (DOCX 15 kb) [file 12916_2015_466_MOESM4_ESM.docx]

**Additional File 4**

**Outcome measurement: pain, peace and grief**

| **Pain (POS pain item)** |
| --- |
| Patient’s experience of pain in the last week of life was measured using a single item from the Palliative care Outcome Scale (POS). The POS is a short easy-to-use clinical outcome measure originally developed and validated in eight end of life and palliative care settings in the UK, including hospital, community, in-patient hospice, outpatient, day care and general practice [1]. The POS pain item measures the overall grade of pain applying verbal descriptors onto a numeric scale with five points (it measures the interference or affect of pain on the patient). Comparisons of patient and family caregiver POS pain ratings show substantial agreement (weighted kappas of 0.69 and 0.61) [2,3]. However, the POS had not been used before in bereavement; our pilot study findings suggested the need for an addition to the question to control for the effect of medication (some respondents were reporting pain despite this being controlled by drugs, with scores reflecting need for medication rather than actual symptom experience) [4]. In addition, the timeframe was changed from the previous three days to the last week of life to fit with the timeframe of measurement in the study.  ***Question*:** Over the last week before s/he died, was s/he affected by pain despite medication?  ***Answer options***: not at all, no effect – pain was completely controlled (0), slightly – but not bothered to get rid of it (1), moderately – pain limited some of her/his ability (2), severely – activities or concentration markedly affected (3), overwhelmingly – s/he was unable to think of anything else (4). |
| **Peace (POS peace item)** |
| Patient’s sense of peace in the last week of life was measured using a single item from the POS family of measures.  The POS peace item measures the overall grade of peace applying verbal descriptors onto a numeric scale with six points (measure of the frequency of patient’s feeling at peace). The item was validated as part of the APCA POS across five palliative care services in South Africa and Uganda [5], although it had not been used with family proxies. No modifications were made following the pilot study, except for the change of timeframe to the last week of life.  ***Question***: Do you think s/he felt at peace, over the last week before s/he died?  ***Answer options***: not at all (0), not very often (1), occasionally (2), some of the time (3), most of the time (4), yes, all the time (5). |
| **Grief (Texas Revised Inventory of Grief – TRIG)** |
| The TRIG is a measure of the intensity of grief experienced by bereaved people. This is a 21-item assessment tool composed of two Likert-type scales, which is easily and rapidly done with minimum intrusion into the bereaved person’s already disrupted life [6]. The items have been developed using factor analysis and the resulting scales have demonstrated reliability, construct and discriminant validity. TRIG has been successfully used clinically and in studies with bereaved relatives of cancer patients [7-9]. The measure was chosen in the process of conducting the pilot study [4], where problems were identified with the previously chosen measure (Core Bereavement Items), and informed by the literature.  ***Scales***  *TRIG I* (Cronbach alpha 0.77 in original validation study, 0.86 in QUALYCARE): 8 first-person statements scored on a five-point scale – completely false (1), mostly false (2), true and false (3), mostly true (4), and completely true (5). The scale measures the intensity of grief around the time of death (“Past Behaviour”), with scores ranging 8 to 40.  *TRIG II* (Cronbach alpha 0.86 in original validation study, 0.93 in QUALYCARE): 13 first-person statements scored on a five-point scale – completely false (1), mostly false (2), true and false (3), mostly true (4), and completely true (5). The scale measures the intensity of grief at time of questionnaire completion (“Present Feelings”), with scores ranging 13 to 65. |

**References**

1. Hearn J, Higginson IJ. Development and validation of a core outcome measure for palliative care: the palliative care outcome scale. Palliative Care Core Audit Project Advisory Group. Qual Health Care 1999; 8(4):219-27.

2. Higginson IJ, Gao W. Caregiver assessment of patients with advanced cancer: concordance with patients, effect of burden and positivity. Health Qual Life Outcomes 2008; 2(6):42.

3. Krug R, Karus D, Selwyn PA, Raveis, VH. Late-stage HIV/AIDS patients' and their familial caregivers' agreement on the palliative care outcome scale. J Pain Symptom Manage 2010; 39(1):23-32.

4. Gomes B, McCrone P, Hall S, Riley J, Koffman J, Higginson IJ. Cognitive interviewing of bereaved relatives to improve the measurement of health outcomes and care utilisation at the end of life in a mortality followback survey. Support Care Cancer 2013; 21(10):2835-44.

5. Harding R, Selman L, Agupio G, Dinat N, Downing J, Gwyther L, et al. Validation of a core outcome measure for palliative care in Africa: the APCA African Palliative Outcome Scale. Health Qual Life Outcomes 2010; 25(8):10.

6. Faschingbauer TR. Texas Revised Inventory of Grief manual. Houston, TX, Honeycomb Publishing Company, 1981.

7. Ringdal GI, Jordhoy MS, Ringdal K, Kaasa S. Factors affecting grief reactions in close family members to individuals who have died of cancer. J Pain Symptom Manage 2001; 22(6):1016-26.

8. Hsieh MC, Huang MC, Lai YL, Lin CC. Grief reactions in family caregivers of advanced cancer patients in Taiwan: relationship to place of death. Cancer Nurs 2007; 30(4):278-84.

9. Grande GE, Ewing G, National Forum for Hospice at Home. Informal carer bereavement outcome: relation to quality of end of life support and achievement of preferred place of death. Palliat Med 2009; 23(3):248-56.
