# Supplementary material for: Is dying in hospital better than home in incurable cancer and what factors influence this? A population-based study
Source: BMC Med. 2015 Oct 9;13:235. doi: 10.1186/s12916-015-0466-5 (PMC4599664; doi:10.1186/s12916-015-0466-5)
Supplement: Additional file 5: — Model of death at home in cancer tested in the QUALYCARE study. (DOCX 14 kb) [file 12916_2015_466_MOESM5_ESM.docx]

**Additional File 5**

**Model of death at home in cancer tested in the QUALYCARE study**

|  | |  |  | |
| --- | --- | --- | --- | --- |
| **Gomes and Higginson’s model (2006)[1]** | |  | **QUALYCARE’s baseline model** | |
|  |  |  |  |  |
| Groups | Factors |  | Independent variables | Source |
|  |  |  |  |  |
| **Illness-related** |  |  |  |  |
|  | non-solid tumours |  | type of cancer (non-solid versus others) | death registration |
|  | long length of disease |  | length of illness | survey questionnaire |
|  | low functional status |  | mobility 3 months before death (EQ-5D) | survey questionnaire |
|  |  |  |  |  |
| **Individual** |  |  |  |  |
| *demographic* | good social conditions |  | patient’s financial hardship | survey questionnaire |
|  | ethnic minorities |  | patient’s country of birth | death registration |
| *personal* | patient’s preferences |  | not entered^a^ | survey questionnaire |
|  |  |  |  |  |
| **Environmental** |  |  |  |  |
| *healthcare input* | use of home care |  | not entered^a^ | survey questionnaire |
|  | intensity of home care |  | GP home visits | survey questionnaire |
|  | availability of inpatient beds |  | n/a^b^ | n/a |
|  | previous admission to hospital |  | hospital days | survey questionnaire |
|  | rural environment |  | n/a^c^ | n/a |
|  | areas of greater hospital provision |  | n/a^b^ | n/a |
| *social support* | living with relatives |  | living with relatives | survey questionnaire |
|  | extended family support |  | number of family caregivers | survey questionnaire |
|  | being married |  | not entered^d^ | survey questionnaire |
|  | caregiver’s preferences |  | not entered^a^ | survey questionnaire |
| *macro-social* | historical trends |  | n/a^e^ | n/a |

^a^ Not entered in multivariate analysis due to numerical problems posed by quasi-separation of the outcome.

^b^ Variable at an area level, with insufficient variation in the study (four health districts within same city).

^c^ Study conducted in urban and suburban environment only.

^d^ Not entered in multivariate analysis to avoid redundancy and multicollinearity with ‘living with relatives’.

^e^ Insufficient variation to study historical trends (study reported to a one-year period).

GP – general practitioner

**Reference**

1. Gomes B, Higginson IJ. Factors influencing death at home for terminally ill patients with cancer: a systematic review. BMJ 2006; 332(7540):515-21.
