# Supplementary material for: Is dying in hospital better than home in incurable cancer and what factors influence this? A population-based study
Source: BMC Med. 2015 Oct 9;13:235. doi: 10.1186/s12916-015-0466-5 (PMC4599664; doi:10.1186/s12916-015-0466-5)
Supplement: Additional file 6: — Recruitment flow diagram. (DOCX 51 kb) [file 12916_2015_466_MOESM6_ESM.docx]

**Additional File 6**

**Recruitment flow diagram**

Eligible (n=1755)

- **Home deaths (n=366)**
- **NHS hospital deaths (n=754)**
- Hospice deaths (n=512)
- Nursing home deaths (n=123)

Sampled (n=1516)

- **Home deaths (n=366)**
- **NHS hospital deaths (n=515)**
- Hospice deaths (n=512)
- Nursing home deaths (n=123)

Questionnaires completed (n=596)

- **Home deaths (n=175)**
- **NHS hospital deaths (n=177)**
- Hospice deaths (n=199)
- Nursing home deaths (n=45)

Questionnaires sent

(n=1516)

**Eligibility assessment**

**Response**

**Analysis**

**Sampling**

Case-control study (n=352)

- **Cases: home deaths (n=175)**
- **Controls: NHS hospital deaths (n=177)**

Assessed for eligibility

(n=7184)

Excluded (n=5429)

- - deaths registered by a coroner (n=2307)
  - age < 18 (n=51)
    - - non-cancer cause of death (n=2940)
        - unspecified place of death (n=20)
- NHS psychiatric hospitals (n=1)
- non-NHS hospitals (n=37)
- residential homes (n=73)

Non-sampled (n=239)

- - NHS hospital deaths (n=239)
    - - District 1 (n=106)
- District 3 (n=133)

Non-respondents (n=920)

- - Active refusals (n=348)
  - Willing but did not complete (n=25)
  - Passed it on but no answer (n=3)
  - Unknown informant status (n=544)
