# Supplementary material for: Is dying in hospital better than home in incurable cancer and what factors influence this? A population-based study
Source: BMC Med. 2015 Oct 9;13:235. doi: 10.1186/s12916-015-0466-5 (PMC4599664; doi:10.1186/s12916-015-0466-5)
Supplement: Additional file 7: — Bivariate analysis of factors associated with home death. (DOCX 24 kb) [file 12916_2015_466_MOESM7_ESM.docx]

**Additional File 7**

**Bivariate analysis of factors associated with home death**

**Table 1. Illness-related factors**

|  | **Home death**  ( n=175) | **Hospital death**  ( n=177) |
| --- | --- | --- |
| Type of cancer (underlying cause of death) ^a^ | **p=0.011** | |
| digestive  respiratory and intra-thoracic organs  eye, brain and other parts of the CNS  breast  lymphoid, haematopoietic and related tissue  genitourinary  unspecified and other | 59 (33.7%)  40 (22.9%)  11 (6.3%)  9 (5.1%)  8 (4.6%)  25 (14.3%)  23 (13.1%) | 46 (26.0%)  40 (22.6%)  2 (1.1%)  9 (5.1%)  18 (10.2%)  42 (23.7%)  20 (11.3%) |
| Mobility at 3 months to death (EQ-5D) | p=0.230 | |
| no problems  some problems  confined to bed | 42 (25.6%)  106 (64.6%)  16 (9.8%) | 53 (31.0%)  105 (61.4%)  13 (7.6%) |
| Self-care at 3 months to death (EQ-5D) | p=0.627 | |
| no problems  some problems  unable to wash/dress her/himself | 68 (42.2%)  68 (42.2%)  25 (15.5%) | 80 (47.1%)  60 (35.3%)  30 (17.6%) |
| Usual activities at 3 months to death (EQ-5D) | p=0.393 | |
| no problems  some problems  unable to perform usual activities | 31 (18.8%)  82 (49.7%)  52 (31.5%) | 45 (26.6%)  70 (41.4%)  54 (32.0%) |
| Length of illness | p=0.629 | |
| less than 6 months  6 months or more, but less than 1 year  1 year or more, but less than 3 years  3 years or more | 55 (32.5%)  33 (19.5%)  54 (32.2%)  27 (16.0%) | 69 (40.1%)  25 (14.5%)  42 (24.4%)  36 (20.9%) |
| Relative’s aware of incurability ≥1week before death | **p<0.001** | |
| never aware or aware for less than 1 week  aware for 1 week or more | 18 (10.7%)  150 (89.3%) | 70 (42.4%)  95 (57.6%) |

^a^ Post-hoc test results: digestive cancers versus others, *p*=0.113; respiratory and intra-thoracic organs versus others *p*=0.954; eye, brain and other parts of CNS versus others, ***p*=0.010**; breast versus others, *p*=0.980; lymphoid, haematopoietic and related tissue, ***p*=0.045**; genitourinary cancers versus others, ***p=*0.024**; unspecified/other versus others, *p*=0.597.

Percentages may not add to 100% due to rounding.

CNS – central nervous system

**Table 2. Individual factors**

|  |  | **Home death**  ( n=175) | **Hospital death**  ( n=177) |
| --- | --- | --- | --- |
| **Demographic** | Patient’s gender | p=0.755 | |
|  | man  woman | 94 (53.7%)  81 (46.3%) | 98 (55.4%)  79 (44.6%) |
|  | Patient’s age | p=0.565 | |
|  | median in years (IQR) | 76 (66-83) | 76 (67-83.5) |
|  | Patient’s country of birth | p=0.744 | |
|  | UK/Ireland  elsewhere | 142 (81.1%)  33 (18.9%) | 146 (82.5%)  31 (17.5%) |
|  | Patient’s ethnicity | p=0.315 | |
|  | white British/Irish  white other/unspecified  other | 145 (84.8%)  13 (7.6%)  13 (7.6%) | 146 (84.4%)  8 (4.6%)  19 (11.0%) |
|  | Patient’s financial hardship | **p=0.035** | |
|  | living comfortably  doing alright on income  just about getting by  finding it difficult or very difficult | 97 (55.7%)  48 (27.6%)  22 (12.6%)  7 (4.0%) | 76 (43.7%)  61 (35.1%)  29 (16.7%)  8 (4.6%) |
|  | Index of multiple deprivation 2010 (patient’s residence area) | **p=0.048** | |
|  | 5^th^ quintile (least deprived)  4^th^ quintile  3^rd^ quintile  2^nd^ quintile  1^st^ quintile (most deprived) | 46 (26.3%)  41 (23.4%)  31 (17.7%)  35 (20.0%)  22 (12.6%) | 43 (24.3%)  28 (15.8%)  24 (13.6%)  50 (28.2%)  32 (18.1%) |
|  |  |  | |
| **Personal** | Patient’s preference for PoD ^a^ | **p<0.001** | |
|  | home  other or no preference | 168 (97.7%)  4 (2.3%) | 94 (66.7%)  47 (33.3%) |
|  | Patient’s discussion of preference for PoD with family | **p<0.001** | |
|  | yes  no | 137 (80.6%)  33 (19.4%) | 65 (39.2%)  101 (60.8%) |
|  | Patient’s discussion of preference for PoD with professionals ^a^ | **p<0.001** | |
|  | yes  no | 109 (76.2%)  34 (23.8%) | 40 (32.3%)  84 (67.7%) |
|  | Patient’s awareness of incurability | **p<0.001** | |
|  | certainly knew  probably knew  probably did not know  definitely did not know | 117 (71.3%)  37 (22.6%)  9 (5.5%)  1 (0.6%) | 63 (40.6%)  56 (36.1%)  22 (14.2%)  14 (9.0%) |
|  | Patient’s religion | p=0.178 | |
|  | Christian  other religion  no religion | 140 (82.8%)  5 (3.0%)  24 (14.2%) | 128 (75.7%)  11 (6.5%)  30 (17.8%) |

^a^ Items with missing data >10% (both with significant differential pattern in missingness by place of death): patient’s preference for PoD (11.1%, 1.7% of home deaths, 20.3% of hospital deaths, ***p*<0.001**), patient’s discussion of preference for PoD with professionals (24.1%, 18.3% of home deaths, 29.9% of hospital deaths, ***p=*0.011**).

Percentages may not add to 100% due to rounding.

IQR – interquartile range; PoD – place of death

**Table 3. Environmental factors**

|  |  | **Home death**  ( n=175) | **Hospital death**  ( n=177) |
| --- | --- | --- | --- |
| **Healthcare input** | Home palliative care use in last 3 months before death ^a^ | **p<0.001** | |
|  | yes  no | 161 (93.6%)  11 (6.4%) | 70 (41.4%)  99 (58.6%) |
|  | Marie Curie nursing use in last 3 months before death | **p<0.001** | |
|  | yes  no | 67 (40.9%)  97 (59.1%) | 7 (4.1%)  163 (95.9%) |
|  | DN/community nursing use in last 3 months before death | **p<0.001** | |
|  | yes  no | 164 (94.8%)  9 (5.2%) | 73 (43.5%)  95 (56.5%) |
|  | Help from home care workers in last 3 months before death | p=0.251 | |
|  | yes  no | 43 (25.4%)  126 (74.6%) | 35 (20.2%)  138 (79.8%) |
|  | Key professional point of contact in last 3 months before death ^b,c^ | **p<0.001** | |
|  | yes  no | 129 (79.1%)  34 (20.9%) | 81 (55.1%)  66 (44.9%) |
|  | GP home visits in last 3 months before death | **p<0.001** | |
|  | 0 or 1 visits  2 visits  3+ visits | 44 (27.0%)  33 (20.2%)  86 (52.8%) | 123 (76.4%)  13 (8.1%)  25 (15.5%) |
|  | Hospital days in last 3 months before death | **p<0.001** | |
|  | 0 to 7 days  8 to 14 days  15 to 28 days  29+ days | 91 (56.5%)  29 (18.0%)  26 (16.1%)  15 (9.3%) | 38 (23.2%)  33 (20.1%)  46 (28.0%)  47 (28.7%) |
|  | Emergency department visits in last 3 months before death | **p<0.001** | |
|  | 0 visits  1 visit  2 visits  3+ visits | 88 (52.4%)  41 (24.4%)  24 (14.3%)  15 (8.9%) | 50 (30.9%)  54 (33.3%)  32 (19.8%)  26 (16.0%) |
|  | Inpatient hospice stay in last 3 months before death | **p=0.002** | |
|  | yes  no | 16 (9.2%)  157 (90.8%) | 3 (1.7%)  170 (98.3%) |
|  | Nursing home stay in last 3 months before death | **p<0.001** | |
|  | yes  no | 0 (0.0%)  172 (100%) | 13 (7.6%)  159 (92.4%) |
|  |  |  | |
| **Social support** | Living with relatives | **p<0.001** | |
|  | yes  no | 145 (83.8%)  28 (16.2%) | 104 (59.4%)  71 (40.6%) |
|  | Family caregivers | **p<0.001** | |
|  | 0 or 1  2 or 3  4+ | 41 (24.3%)  72 (42.6%)  56 (33.1%) | 69 (40.4%)  64 (37.4%)  38 (22.2%) |
|  | Patient’s marital status | **p=0.001** | |
|  | married/with partner  widowed  divorced/separated  never married | 117 (68.8%)  34 (20.0%)  9 (5.3%)  10 (5.9%) | 82 (47.4%)  52 (30.1%)  16 (9.2%)  23 (13.3%) |
|  | Relative’s gender | p=0.755 | |
|  | man  woman | 53 (30.6%)  120 (69.4%) | 65 (36.9%)  111 (63.1%) |
|  | Relative’s age | p=0.288 | |
|  | median in years ( IQR) | 60 (50-71) | 57 (49-68) |
|  | Relative’s relationship to patient | **p<0.001** | |
|  | spouse/partner  son/daughter  brother/sister  other | 85 (48.6%)  75 (42.9%)  4 (2.3%)  11 (6.3%) | 62 (35.2%)  67 (38.1%)  16 (9.1%)  31 (17.6%) |
|  | Relative’s preference for PoD at 3 months before death | **p<0.001** | |
|  | home  other or no preference | 159 (91.9%)  14 (8.1%) | 56 (32.0%)  119 (68.0%) |
|  | Change in relative’s preference for PoD in 3 months prior death | p=0.07 | |
|  | yes  no | 16 (9.5%)  153 (90.5%) | 26 (16.0%)  136 (84.0%) |
|  | Relative’s disagreement with patient’s preference for PoD ^c^ | **p<0.001** | |
|  | yes  no | 162 (94.2%)  10 (5.8%) | 96 (71.6%)  38 (28.4%) |
|  | Relative’s work arrangements in last 3 months before death |  | |
|  | 0 to 3 days off work  4 to 14 days off work  15+ days off work  not working ^d^ | 12 (7.3%)  26 (15.9%)  34 (20.7%)  92 (56.1%) | 48 (29.4%)  22 (13.5%)  14 (8.6%)  79 (48.5%) |
|  | Relative’s presence at time of death | **p<0.001** | |
|  | yes  no | 153 (88.4%)  20 (11.6%) | 96 (54.2%)  81 (45.8%) |

^a^ This included palliative care teams, Macmillan nurses, staff described as hospice or palliative care by the respondents.

^b^ Key professional point of contact was: a nurse (34.3%), GP (23.0%), another doctor (10.3%), other professional (17.2%), 2+ professionals (15.2%).

^c^ Items with missing data >10% (both with significant differential pattern in missingness by place of death): key professional point of contact in last 3 months before death (11.9%, 6.9% of home deaths, 16.9% of hospital deaths, ***p*=0.003**), relative’s disagreement with patient’s preference for PoD (13.1%, 1.7% of home deaths, 24.3% of hospital deaths, ***p*<0.001**).

^d^ Due to retirement in 79.5% of the cases or to unemployment in 17.5% (in addition, 2 relatives were studying and 3 were not working for other reasons not specified).

Percentages may not add to 100% due to rounding.

DN – district nurse; GP – general practitioner; PoD – place of death
