# Supplementary material for: Is dying in hospital better than home in incurable cancer and what factors influence this? A population-based study
Source: BMC Med. 2015 Oct 9;13:235. doi: 10.1186/s12916-015-0466-5 (PMC4599664; doi:10.1186/s12916-015-0466-5)
Supplement: Additional file 8: — Sub-analysis of five-factor model with patients who preferred to die at home. (DOCX 16 kb) [file 12916_2015_466_MOESM8_ESM.docx]

**Additional File 8**

**Sub-analysis of five-factor model with patients who preferred to die at home**

This sub-group analysis tested the final model of factors associated with home death only for patients who preferred to die at home (Table 1). It aimed to help clinicians make decisions when caring for patients they know wish to die at home. The results were compared with those from all patients (regardless of where they preferred to die); these are presented in the paper. All factors remained significant except for discussion of patient preference for place of death with family, which lost strength and significance once adjusted (OR 3.33, 95%CI 1.88 to 5.86; AOR 2.21, 95%CI 0.95 to 5.16). Compared to the results for all patients, the model still explained most variance in place of death but slightly less (51.9% compared to 59.2%); it classified less well cases of death in hospital (61.4% as opposed to 78.9%), but more correctly cases of home death (84.4% as opposed to 82.1%), with an overall success rate of 76.6% (compared to 80.6%).

**Table 1. Direct logistic regression home vs. hospital death: patients with home preference (*n*=205)**

|  |  | Unadjusted |  |  |  | Adjusted |  |
| --- | --- | --- | --- | --- | --- | --- | --- |
|  | *n* | OR (95%CI) | *p* |  | *n* | OR (95%CI) | *p* |
| **Length of relative’s awareness of incurability** |  |  | **<0.001** |  |  |  | **<0.001** |
| never or aware for less than one week | 53 | *Ref.* |  |  | 44 | *Ref.* |  |
| aware for one week or more | 200 | 5.13 (2.68 to 9.80) |  |  | 161 | 6.55 (2.78 to 16.97) |  |
| **Patient’s preference for place of**  **death discussed with family** |  |  | **<0.001** |  |  |  | 0.068 |
| no | 71 | *Ref.* |  |  | 58 | *Ref.* |  |
| yes | 186 | 3.33 (1.88 to 5.86) |  |  | 147 | 2.21 (0.95 to 5.16) |  |
| **Hospital days** |  |  | **<0.001** |  |  |  | **0.002** |
| 0 to 7 | 111 | *Ref.* |  |  | 92 | *Ref.* |  |
| 8 to 14 | 47 | 0.42 (0.20 to 0.89) |  |  | 40 | 0.64 (0.20 to 2.00) |  |
| 15 to 28 | 46 | 0.29 (0.14 to 0.60) |  |  | 39 | 0.34 (0.12 to 0.97) |  |
| 29+ | 39 | 0.16 (0.07 to 0.36) |  |  | 34 | 0.12 (0.04 to 0.37) |  |
| **GP home visits** |  |  | **<0.001** |  |  |  | **<0.001** |
| 0 or 1 visit | 110 | *Ref.* |  |  | 89 | *Ref.* |  |
| 2 visits | 37 | 8.05 (3.10 to 20.91) |  |  | 31 | 4.85 (1.37 to 17.11) |  |
| 3+ visits | 99 | 8.08 (4.19 to 15.01) |  |  | 85 | 6.86 (2.78 to 16.97) |  |
| **Relative’s work arrangements**  **in 3 months before death** |  |  | **<0.001** |  |  |  | **0.042** |
| 0 to 3 days off work | 38 | *Ref.* |  |  | 28 | *Ref.* |  |
| 4 to 14 days off work | 38 | 3.71 (1.44 to 9.06) |  |  | 36 | 2.55 (0.65 to 1.00) |  |
| 15+ days of work | 40 | 8.67 (3.08 to 24.36) |  |  | 37 | 8.84 (1.86 to 41.94) |  |
| not working | 126 | 5.21 (2.38 to 11.42) |  |  | 104 | 3.70 (1.13 to 12.11) |  |

Analysis conducted with the sub-group of patients who preferred to die at home. Multivariate model included 205 cases (ratio of 34:1 cases per variable), and adjusted for health district (*p*=0.322). Model statistics: Nagelkerke R^2^=0.519, Hosmer and Lemeshow *X*^2^(8,205)=8.976, *p*=0.344. Correctly predicted 84.4% of home deaths and 61.4% of hospital deaths, with an overall success rate of 76.6%.

CI – confidence interval; GP – general practitioner; OR – odds ratio; Ref. – reference category
