# Supplementary material for: Is dying in hospital better than home in incurable cancer and what factors influence this? A population-based study
Source: BMC Med. 2015 Oct 9;13:235. doi: 10.1186/s12916-015-0466-5 (PMC4599664; doi:10.1186/s12916-015-0466-5)
Supplement: Additional file 9: — Sensitivity analysis of non-participation on factors associated with home death. (DOCX 21 kb) [file 12916_2015_466_MOESM9_ESM.docx]

**Additional File 9**

**Sensitivity analysis of non-participation on factors associated with home death**

This sensitivity analysis examined the impact of non-response on the association of five variables with home death (those available through death registrations): patient’s gender and age, country of birth, cancer type and deprivation. It compared direct logistic regression results obtained from the whole sample (participants and non-participants, Table 1) with those from participants only (Table 2).

Both analyses found that type of cancer was the only factor significantly associated with home death. Patients with non-solid tumours (i.e. lymphoid, haematopoietic and related tissue) were less likely to die at home than patients with digestive cancer (AOR 0.27, 95%CI 0.10 to 0.71 amongst participants and AOR 0.44, 95% 0.25 to 0.76 in the whole sample). Patients with genitourinary cancer were also less likely to die at home than patients with digestive cancer, but only amongst participants (AOR 0.46, 95%CI 0.23 to 0.90), not in the whole sample (AOR 0.71, 95%CI 0.46 to 1.08). The influence of deprivation was not significant, both in the whole sample (*p*=0.051) and amongst participants (*p*=0.273). The variance in place of death accounted for these models (based solely on death registration data) was small (Nagelkerke R^2^ 0.14 amongst participants and 0.07 in the whole sample).

**Table 1. Direct logistic regression home vs. hospital death: whole sample (*n*=877)**

|  | *n* |  | Unadjusted |  |  | Adjusted |  |
| --- | --- | --- | --- | --- | --- | --- | --- |
|  |  |  | OR (95%CI) | *p* |  | OR (95%CI) | *p* |
| **Type of cancer** |  |  |  | **0.010** |  |  | **0.011** |
| digestive | 240 |  | *Ref.* |  |  | *Ref.* |  |
| genitourinary | 161 |  | 0.72 (0.48 to 1.09) |  |  | 0.71 (0.46 to 1.08) |  |
| non-solid | 84 |  | 0.47 (0.27 to 0.81) |  |  | 0.44 (0.25 to 0.76) |  |
| other/unspecified | 392 |  | 1.04 (0.75 to 1.43) |  |  | 0.97 (0.69 to 1.36) |  |
| **Gender** |  |  |  | 0.307 |  |  | 0.225 |
| man | 455 |  | *Ref.* |  |  | *Ref.* |  |
| woman | 422 |  | 1.15 (0.88 to 1.50) |  |  | 1.19 (0.90 to 1.59) |  |
| **Age** |  |  |  | 0.509 |  |  | 0.291 |
| 20-49 | 39 |  | *Ref.* |  |  | *Ref.* |  |
| 50-59 | 79 |  | 1.08 (0.49 to 2.36) |  |  | 0.99 (0.44 to 2.23) |  |
| 60-69 | 152 |  | 1.46 (0.72 to 2.99) |  |  | 1.35 (0.64 to 2.85) |  |
| 70-79 | 272 |  | 1.27 (0.64 to 2.52) |  |  | 1.20 (0.59 to 2.46) |  |
| 80-89 | 288 |  | 1.14 (0.58 to 2.26) |  |  | 1.02 (0.50 to 2.07) |  |
| 90+ | 47 |  | 0.78 (0.32 to 1.90) |  |  | 0.61 (0.25 to 1.51) |  |
| **Country of birth** |  |  |  | **0.026** |  |  | 0.130 |
| UK/Ireland | 703 |  | *Ref.* |  |  | *Ref.* |  |
| overseas | 174 |  | 0.68 (0.48 to 0.95) |  |  | 0.75 (0.52 to 1.09) |  |
| **IMD 2010** |  |  |  | **0.001** |  |  | 0.051 |
| 5^th^ quintile (least deprived) | 181 |  | *Ref.* |  |  | *Ref.* |  |
| 4^th^ quintile | 164 |  | 1.01 (0.66 to 1.54) |  |  | 1.02 (0.66 to 1.58) |  |
| 3^rd^ quintile | 143 |  | 0.79 (0.51 to 1.22) |  |  | 0.97 (0.60 to 1.57) |  |
| 2^nd^ quintile | 211 |  | 0.52 (0.34 to 0.78) |  |  | 0.58 (0.37 to 0.91) |  |
| 1^st^ quintile (most deprived) | 178 |  | 0.52 (0.34 to 0.80) |  |  | 0.58 (0.33 to 1.03) |  |

Multivariate model included 877 cases with no missing data (ratio 146:1 cases per variable), and adjusted for health district (*p*=0.194). Model statistics: Nagelkerke R^2^=0.070, Hosmer and Lemeshow *X*^2^(8, 877)=4.320, *p*=0.827. Correctly predicted 37.0% of home deaths and 79.0% of hospital deaths, with an overall success rate of 61.3%.

CI – confidence interval; IMD – index of multiple deprivation; OR – odds ratio; Ref. – reference category

**Table 2. Direct logistic regression home vs. hospital death: participants only (*n*=352)**

|  | *n* |  | Unadjusted |  |  | Adjusted |  |
| --- | --- | --- | --- | --- | --- | --- | --- |
|  |  |  | OR (95%CI) | *p* |  | OR (95%CI) | *p* |
| **Type of cancer** |  |  |  | **0.015** |  |  | **0.013** |
| digestive | 105 |  | *Ref.* |  |  | *Ref.* |  |
| genitourinary | 67 |  | 0.46 (0.25 to 0.87) |  |  | 0.46 (0.23 to 0.90) |  |
| non-solid | 26 |  | 0.35 (0.14 to 0.87) |  |  | 0.27 (0.10 to 0.71) |  |
| other/unspecified | 154 |  | 0.91 (0.55 to 1.50) |  |  | 0.83 (0.48 to 1.43) |  |
| **Gender** |  |  |  | 0.756 |  |  | 0.528 |
| man | 192 |  | *Ref.* |  |  | *Ref.* |  |
| woman | 160 |  | 1.07 (0.70 to 1.63) |  |  | 1.16 (0.73 to 1.86) |  |
| **Age** |  |  |  | 0.178 |  |  | 0.127 |
| 20-49 | 15 |  | *Ref.* |  |  | *Ref.* |  |
| 50-59 | 26 |  | 0.49 (0.13 to 1.78) |  |  | 0.33 (0.08 to 1.32) |  |
| 60-69 | 70 |  | 0.63 (0.20 to 1.96) |  |  | 0.42 (0.13 to 1.43) |  |
| 70-79 | 101 |  | 0.74 (0.24 to 2.22) |  |  | 0.50 (0.15 to 1.64) |  |
| 80-89 | 111 |  | 0.78 (0.26 to 2.35) |  |  | 0.54 (0.17 to 1.77) |  |
| 90+ | 29 |  | 0.25 (0.68 to 0.95) |  |  | 0.17 (0.04 to 0.69) |  |
| **Country of birth** |  |  |  | 0.744 |  |  | 0.408 |
| UK/Ireland | 288 |  | *Ref.* |  |  | *Ref.* |  |
| overseas | 64 |  | 1.10 (0.64 to 1.88) |  |  | 1.30 (0.70 to 2.41) |  |
| **IMD 2010** |  |  |  | 0.097 |  |  | 0.273 |
| 5^th^ quintile (least deprived) | 89 |  | *Ref.* |  |  | *Ref.* |  |
| 4^th^ quintile | 69 |  | 1.37 (0.73 to 2.58) |  |  | 1.48 (0.74 to 2.94) |  |
| 3^rd^ quintile | 55 |  | 1.21 (0.61 to 2.37) |  |  | 1.61 (0.75 to 3.48) |  |
| 2^nd^ quintile | 85 |  | 0.65 (0.36 to 1.19) |  |  | 0.96 (0.43 to 1.72) |  |
| 1^st^ quintile (most deprived) | 54 |  | 0.64 (0.32 to 1.27) |  |  | 0.67 (0.26 to 1.74) |  |

Multivariate model included 352 cases with no missing data (ratio 59:1 cases per variable), and adjusted for health district (*p*=0.023). Model statistics: Nagelkerke R^2^=0.135, Hosmer and Lemeshow *X*^2^(8,352)=4.208, *p*=0.838. Correctly predicted 66.3% of home deaths and 59.9% of hospital deaths, with an overall success rate of 63.1%.

CI – confidence interval; IMD – index of multiple deprivation; OR – odds ratio; Ref. – reference category
